# HIV-affected couples and individuals who desire children should be offered options for safer conception

**DOI:** 10.7448/IAS.20.1.22155

**Published:** 2017-07-26

**Authors:** Lealah C Pollock, Shannon Weber, Angela Kaida, Lynn T Matthews, Dominika L Seidman

**Affiliations:** ^a^ Family and Community Medicine, University of California San Francisco, San Francisco, CA, USA; ^b^ Faculty of Health Sciences, Simon Fraser University, Burnaby, Canada; ^c^ Division of Infectious Disease, Massachusetts General Hospital, Boston, MA, USA; ^d^ Obstetrics, Gynecology & Reproductive Sciences, University of California San Francisco, San Francisco, CA, USA

**Keywords:** HIV, human immunodeficiency virus, natural conception, serodiscordant, patient-centred counselling, safer conception

Safier and Sauer argue that assisted reproductive technologies should be recommended as the first-line conception strategy for HIV-affected couples desiring children [1]. We agree with honouring the reproductive goals and preferences of individuals and couples affected by HIV. However, given the evidence that HIV-affected couples can achieve pregnancy through condomless sex with zero or near-zero risk of HIV transmission [2], we disagree with the authors’ assertion that assisted reproductive technologies should be the treatment of choice over other safer conception strategies. Women and men living with HIV desire counselling on a range of safer conception options; offering this counselling will allow individuals to meet their reproductive goals while minimizing HIV transmission [3,4].

Research demonstrates that “condomless sex” does not necessarily indicate “unprotected sex”: sustained use of antiretroviral therapy (ART) to achieve a suppressed viral load in partners living with HIV, known as treatment as prevention (TasP), is associated with zero or near-zero risk of sexual HIV transmission [5,6]. Indeed, there are no reported cases of penile–vaginal transmission in the setting of an undetectable viral load [7]. Both prospective cohort and modelling studies suggest that antiretroviral therapy for partners living with HIV combined with genital tract infection screening and condomless sex limited to peak fertility maximizes the likelihood of pregnancy while minimizing, or eliminating, sexual HIV transmission [8,9]. For individuals or couples who desire additional protection, or when partners living with HIV take ART inconsistently, pre-exposure prophylaxis (PrEP) offers a safe and highly effective method to reduce HIV acquisition risk while allowing for conception [10].

As noted in the editorial accompanying the recent supplement, “healthcare providers should support informed, voluntary decision-making about reproductive choices, to create environments that reduce stigma associated with HIV” [11]. With multiple safe and effective conception options available, providers have an obligation to inquire about each person’s values and preferences and provide counselling that supports informed decision-making. Promoting only one option imposes counsellors’ preferences over couples’ preferences, which might include prioritizing relationship intimacy or minimizing interventions. Eliciting and respecting these preferences engages patients in shared decision-making [12].

Finally, Safier and Sauer argue that there are two strata of HIV-affected couples: those who can afford and access the “preferred treatment option” and those who cannot. Reproductive health counselling must be grounded in a reproductive justice framework: options for safer conception should be available to *all* HIV-affected individuals and couples. Assisted reproductive technologies are essential when spontaneous conception is not possible, such as for same sex couples, single parents by choice and couples affected by infertility. In addition, some HIV-affected couples may prefer to use assisted reproductive technologies for all of the reasons outlined by Safier and Sauer. Infertility specialists have played a critical role in supporting the reproductive rights of persons living with HIV [13]. They will continue to do so by making safe and effective services available to clients who need and want them. All providers caring for people affected by HIV can support their patients by counselling about reproductive options in a client-centred, evidence-based, and rights-based manner.

## Abbreviations

HIV, Human Immunodeficiency Virus; TaSP, Treatment as Prevention; PrEP, Pre-Exposure Prophylaxis.
